# The Influence of Arrhythmias and Metabolic Profile on Inpatient Mortality in Patients with Left Ventricular Assist Devices

**DOI:** 10.3390/jcm13061737

**Published:** 2024-03-17

**Authors:** Daniel Antwi-Amoabeng, Bryce David Beutler, Tokunbo David Gbadebo

**Affiliations:** 1 Department of Medicine, CHRISTUS Ochsner St. Patrick Hospital, Lake Charles, LA 70602, USA; 2 Department of Radiology, Keck School of Medicine, University of Southern California, Los Angeles, CA 90033, USA; 3 Cardiology Section, Emory Decatur Hospital, Decatur, GA 30033, USA

**Keywords:** left ventricular assist device, mortality, arrhythmia, heart failure, hyperkalemia, metabolic disturbance

## Abstract

**Background:** In patients with end-stage heart failure, durable Left Ventricular Assist Devices (LVADs) can be used as a bridge to transplant or destination therapy. LVADs have been shown to improve survival for patients with heart failure (HF). HF is associated with electrolyte abnormalities and the development of sustained arrhythmias. However, data on the influence of arrhythmias and electrolyte imbalances on inpatient outcomes in LVAD patients are lacking. Furthermore, previous works assessing inpatient outcomes focused mainly on the role of chronic comorbidities in those outcomes. **Methods:** In this cross-sectional study, we used discharge data from the National Inpatient Sample from 2019 to 2020 to assess the influence of acute arrhythmias on inpatient mortality in patients with LVADs. We also investigated the relationship between acute medical conditions and mortality. **Results:** There were 9418 (not survey-adjusted) hospitalizations with LVAD, among which 2539 (27%) died during the hospitalization. Univariate analysis of arrhythmias showed that ventricular arrhythmias (VAs)—ventricular fibrillation/flutter and ventricular tachycardia—as well as complete heart block were associated with significantly higher odds of mortality. Follow-up multivariable logistic analysis showed that these arrhythmias retain their increased association with death. Hyperkalemia and acidosis had increased adjusted odds of death (1.54 (95% confidence interval: 1.28–1.85) (*p* < 0.001) and 2.44 (CI: 2.14–2.77) (*p* < 0.001), respectively). **Conclusions:** VAs, complete heart block, hyperkalemia, and acidosis were associated with increased odds of all-cause mortality. Females had higher odds of inpatient mortality. These findings suggest that electrolyte management, maintenance of optimal acid–base balance, and interventions to treat sustained ventricular arrhythmias may be suitable therapeutic targets to reduce mortality in hospitalized patients with LVADs.

## 1. Introduction

Durable Left Ventricular Assist Devices (LVADs) are an alternative to heart transplants in patients with end-stage heart failure. In addition, LVADs can be used as a bridge to transplant, a bridge to candidacy, or a destination therapy in some patients and have been shown to improve survival for patients with heart failure (HF). There has been a recent increase in the number of LVAD implantations in the United States; the highest number of implantations in history occurred in 2019 per the Society of Thoracic Surgeons Interagency Registry for Mechanical Assisted Circulatory Support 2020 annual report [1]. However, despite advances in LVAD technology, major bleeding, infection, and acute heart failure continue to be leading causes of hospitalization and death among LVAD patients [2,3]. Preimplantation comorbid conditions and patient sociodemographic characteristics have been shown to affect mortality in LVAD patients.

Post-implantation electrical remodeling may create myocardial substrates susceptible to arrhythmias. Ventricular arrhythmias (VAs) may be triggered by this mechanism or by LVAD dysfunction, such as a suck-down event in which there is a precipitous decline in LVAD output due to direct contact between the inflow cannula and the ventricular wall or septum [4]. VAs, in turn, can result in low-flow events and hemodynamic compromise [5]. Atrial arrhythmias (AAs), in contrast, can lead to thromboembolism [6], and when accompanied by high ventricular response, may cause progressive hemodynamic compromise of the right heart with a resultant reduction in preload delivery to the LVAD [7]. AAs and VAs therefore both present an increased risk of morbidity and mortality in LVAD patients.

Neurohormonal responses to renal hypoperfusion and diuretics used in HF often lead to electrolyte derangements, renal dysfunction, and acid–base imbalance [8]. The resultant abnormalities in serum potassium, magnesium, phosphorus, and calcium have been shown to have a strong association with atrial and ventricular arrhythmias [9,10,11]. Furthermore, metabolic acidosis can lead to alterations in cellular electrophysiology, which may increase the risk of post-acidosis arrhythmias [12]. However, there is a paucity of data on the influence of the metabolic milieu and incident arrhythmia on inpatient mortality among hospitalized patients with LVAD. In this cross-sectional study, we catalog the distribution of arrhythmias among patients hospitalized with LVADs. In addition, we assess the influence of the interplay between electrolyte disturbances and arrhythmias on inpatient mortality. As noted by the STS-INTERMACS (The Society of Thoracic Surgeons (STS) Interagency Registry for Mechanically Assisted Circulatory Support (Intermacs) 2022 annual report), there has been a shift in the indication for LVADs in recent years, with 81.1% of implantations for destination therapy [13]. Therefore, hospital encounters involving LVAD patients are expected to increase. Understanding risks for inpatient mortality in LVAD patients will allow clinicians to minimize exposure to acute risk factors and plan appropriate corrective interventions during the clinical course of hospitalized patients with LVADs.

## 2. Methods

### 2.1. Study Population

We used the International Classification of Diseases, Tenth Revision, Procedure Coding System (ICD-10-PCS codes) to extract encounters involving the presence of LVADs from the National Inpatient Sample (NIS) Healthcare Cost and Utilization Project (HCUP), Agency for Healthcare Research and Quality (AHRQ) database year 2019 to 2020. The NIS is a publicly available survey database that systematically samples discharge encounters from approximately 20% of all hospitalizations in the United States. It is the largest database of its kind and estimates nationwide and regional inpatient healthcare utilization and outcomes in the United States. Since data from the NIS is de-identified, approval from our institutional review board was not required for this study.

We used ICD-10-CM codes to identify encounters with acute heart failure and/or acute exacerbation of chronic heart failure, supraventricular and ventricular arrhythmias, as well as conduction abnormalities at any diagnosis coding position. We used the following codes: supraventricular tachycardia (SVT): ‘I471’, ventricular tachycardia (VT): ‘I472’, ventricular tachycardia (VT): ‘I4901’, ventricular fibrillation/flutter (VF): ‘I4902’, atrial fibrillation/flutter (AF): ‘I480’ ‘I481’ ‘I4811’ ‘I4819’ ‘I482’ ‘I4820’ ‘I4821’ ‘I4891’ ‘I483’ ‘I484’ ‘I4892’, Long QT syndrome: ‘I4581’, sick sinus syndrome (SSS): ‘I495’, first degree A-V block: ‘I440’, second degree A-V block: ‘I441’, complete heart block (CHB): ‘I442’, left bundle branch block (LBBB): ‘I447’, right bundle branch block (RBBB): ‘I450’ ‘I4510’ ‘I4519’, and fascicular blocks: ‘I444’ ‘I445’ ‘I4460’ ‘I4469’ ‘I450’ ‘I452’ ‘I453’ to extract the frequency of arrhythmias coded in the discharge data from the National Inpatient Sample database from 2019 to 2020. Codes for acute heart failure and LVAD were previously described [14,15]. Encounters with ages less than 18 years old and observations with missing data for age, sex, race, and inpatient mortality were excluded from the analysis. We extracted lifestyle habits, comorbid medical conditions, and patient demographic information and included these in covariate analyses.

### 2.2. Statistical Analyses

Continuous variables were reported as median (interquartile range) and categorical variables as counts (percentage of the study sample). We compared the differences in the distribution of patient demographics, chronic comorbid conditions, arrhythmias, and inpatient acute events between subjects who died and those who did not use the Chi-squared test for categorical variables and the Wilcoxon Rank Sum test for equality of the means of continuous variables and show the results in Table 1. We used the Charlson comorbidity index to compare the burden of predefined chronic conditions between the groups. The comorbidities were based on definitions provided within the NIS database and broadly classified as a composite of varying disease severity; comorbid liver disease and chronic pulmonary disease, for example, included patients with both mild and severe illness. ICD-10 codes were used to identify additional chronic comorbid conditions and acute inpatient events. In univariate logistic regression analysis, we assessed the odds of death using the variables presented in Table 1 and generated a covariate matrix. We included covariates with statistical significance or those deemed to have clinical significance in a base multivariable logistic regression to model the predictors of inpatient mortality. The most parsimonious model was selected by stepwise elimination of covariates. Model fitness was assessed with appropriate post-estimation tests. Analyses were survey-weighted to account for the nature of the NIS data where appropriate. All analyses were performed at a two-tailed 5% level of significance using Stata version 16.1 (Stata Corporation, College Station, TX, USA).

## 3. Results

### 3.1. Baseline Characteristics

The study included 11,292,838 (56,464,192 when survey weights are applied) hospitalizations over the two years from January 2019 through December 2020. Of these, 9418 (47,090 survey-weighted) (0.08%) had LVAD, a vast majority of whom were males (6784 (72%)). The median age of the LVAD cohort was 67 years (interquartile range: 58–75 years). Females were older than males (68 (58–77) years vs. 67 (57–75) years). Most of the subjects were in the 61–70-year age group. The median age of those who died was 68 (60–76) years, which was significantly older than those who survived 66 (57–75) (*p*-value < 0.001). Whites constituted the majority (70.5%) whereas Native Americans were the least represented in the cohort (0.9%). Chronic comorbid conditions grouped by the Charlson comorbidity index showed that a majority of the subjects had 2 or more of the indexed comorbid conditions. Table 1 summarizes the baseline patient demographics and comorbid conditions.

### 3.2. Acute Inpatient Events

There was no difference in the event rates of acute ischemic and hemorrhagic stroke between those who died and those who survived. A similar observation exists for those who had a non-ST-elevation myocardial infarction (NSTEMI), deep vein thrombosis (DVT), or pulmonary embolism (PE). Among patients who died, kidney injury was the most common acute inpatient event; the incidence rate was 1745 (68.7%), which was significantly higher in this group (*p*-value < 0.001) as compared to those who did not die 3363 (48.9%). Apart from acute heart failure, where significantly more subjects survived the hospital stay, all other acute events that showed significant differences in the distribution occurred more frequently in patients who died. These include those who required invasive mechanical ventilation and those who had STEMI, kidney and liver injury, gastrointestinal bleeding, sepsis, and septic shock.

We used volume status, acid–base balance status, and electrolyte abnormalities as surrogate measures of metabolic states that may have existed during the hospitalization. There was no statistically significant difference in the volume status denoted by either volume overload or dehydration between those who died and those who survived. Similarly, electrolyte abnormalities involving calcium, magnesium, and sodium as well as alkalotic state did not show a significant difference in distribution between the groups. However, disturbances in the serum concentration of potassium appear to have an association with inpatient mortality. Hyperkalemia was more prevalent in subjects who died 405 (15.9%) compared to those who survived 588 (8.5%) (*p*-value < 0.001). Acidosis was also more common among those who died: 1310 (51.6%) versus 1510 (21.9%) (*p*-value < 0.001).

### 3.3. Burden of Arrhythmias

**Figure 1:**
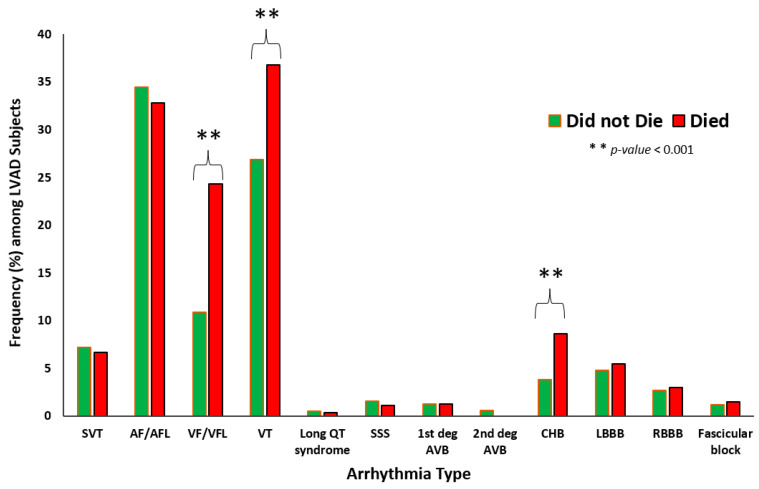
Comparative distribution of arrhythmias between subjects with LVAD who died versus those who survived the hospitalization. SVT: supraventricular tachycardia; AF/AFL: atrial fibrillation/flutter; VF/VFL: ventricular fibrillation/flutter; SSS: sick sinus syndrome; AVB: atrioventricular block; CHB: complete heart block; LBBB: left bundle branch block; RBBB: right bundle branch block.

AF was the most common arrhythmia, occurring in 3203 (34%) of subjects, followed by VT (2787 (29.6%) subjects) and VF (1367 (14.5%) subjects). Long QT syndrome was the least frequent arrhythmia; only 45 (0.5%) of the subjects had this dysrhythmia. The frequency distribution of incident arrhythmia among those who died compared to those who survived is shown in Figure 1. A significantly higher proportion of the subjects who died had VT, VF, and complete heart block as compared to those who did not: VF (617 (24.3%) died compared to 750 (10.9%) of those who did not die (*p*-value < 0.001)), VT (died = 933 (36.8%) versus alive = 1854 (26.9%) (*p*-value < 0.001)), and for CHB (died = 218 (8.6%) versus alive = 259 (3.8%) (*p*-value < 0.001)).

### 3.4. Predictors of All-Cause Mortality

**Figure 2:**
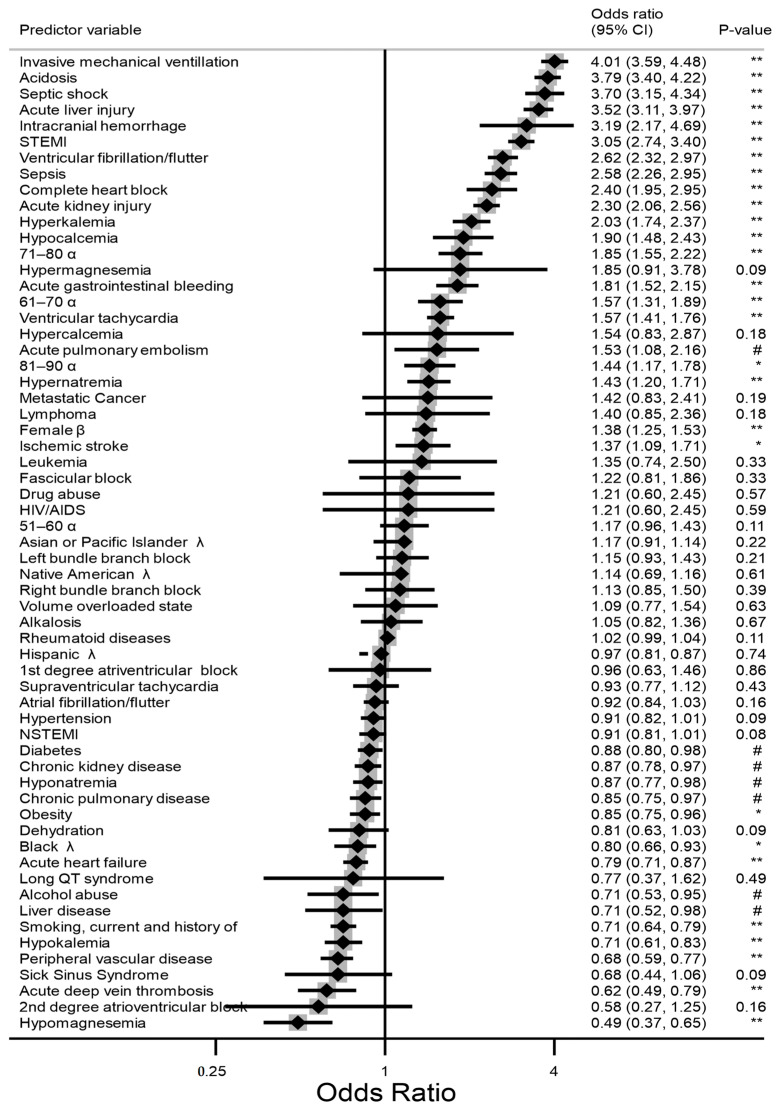
Forest plot of the unadjusted odds of mortality among hospitalizations with LVAD. Key: α = reference age group:18–50 years; β = compared to males; λ = compared to whites. # = *p*-value < 0.05, * = *p*-value < 0.01; ** = *p*-value < 0.001.

The inpatient mortality rate for the LVAD cohort was 27%, which was significantly more than that of the no-LVAD sample (2.7%); the *p*-value for the test of equality of proportions was <0.001. Initial univariate analysis showed that invasive mechanical ventilation had the strongest association with inpatient mortality in our cohort (odds ratio (OR) = 4.01 (95% confidence interval: 3.59–4.48). The occurrence of sepsis, septic shock, acute liver injury, acute kidney injury, intracranial hemorrhage, ischemic stroke, STEMI, acute GI bleeding, and acute pulmonary embolism were associated with increased odds of death. Among the metabolic derangements, acidosis, hyperkalemia, hypocalcemia, and hypernatremia were all associated with increased odds of death. VF, VT, and complete heart block were the only arrhythmias with ORs greater than 1. Age over 60 years and female sex showed increased odds of death. Neither race nor chronic medical conditions had a significant association with death. The results of the initial univariate logistic regression analysis are shown in Figure 2. All data in Figure 2 were identified using predefined comorbidities in the NIS database or ICD-10 codes when conditions were not available in the predefined test.

**Figure 3:**
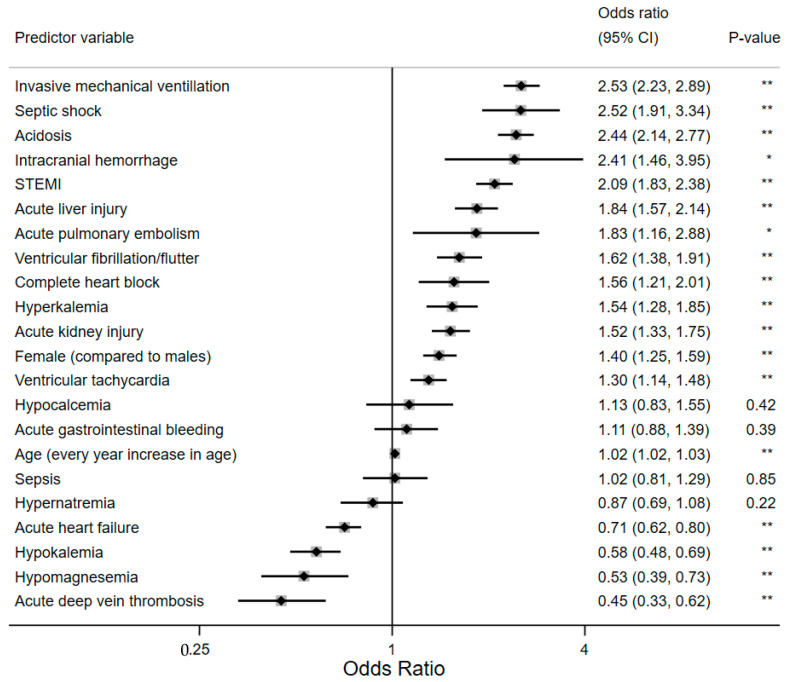
Forest plot showing the predictors of in-patient mortality among subjects with LVAD in multivariable logistic regression analysis. Key: * = *p*-value < 0.01; ** = *p*-value < 0.001.

Multivariable logistic regression analysis showed that the three rhythm disturbances retained their significant association with increased odds of mortality when odds ratios were adjusted for covariates. VF had the highest adjusted odds ratio (aOR) of 1.62 (CI: 1.38–1.91) (*p* < 0.001), and VT had the lowest aOR of 1.3 (1.14–1.48) (*p* < 0.001). Overall, invasive mechanical ventilation had the highest odds 2.53 (2.23–2.89) (*p* < 0.001). Other inpatient events that showed statistically significant increased odds of mortality include septic shock, intracranial hemorrhage, acute liver injury, STEMI, acute pulmonary embolism, and acute kidney injury. Hyperkalemia and acidosis were the only metabolic disturbances that retained increased odds of mortality; 1.54 (1.28–1.85, *p* < 0.001), and 2.44 (2.14–2.770, *p* < 0.001), respectively. The odds of death in females were 1.4 times higher than in males (95% CI: 1.25–1.59) (*p* < 0.001), and for every unit increase in age, the odds of inpatient mortality were 1.02 times higher. Figure 3 is a forest plot of the adjusted odds of mortality in this LVAD cohort.

## 4. Discussion

LVADs were first developed in the 1960s and have since evolved from a temporizing measure to maintain cardiac output in patients with severe myocardial dysfunction to a mainstay of HF management [16]. The current generation of LVADs plays a critical role as a bridge to recovery in patients with impaired left ventricular function, including those with transitory cardiomyopathy and individuals who cannot be successfully weaned from cardiopulmonary bypass. In addition, LVADs may serve as a bridge to heart transplantation in eligible patients or as a destination therapy in patients who are not deemed transplant candidates [17]. The preponderance of the evidence has established that LVADs improve quality of life and survival [18]. However, despite advances in device technology and improved surgical techniques, mortality remains relatively high; patients requiring an LVAD as a bridge to recovery or heart transplantation face a mortality rate of approximately 25 to 40% whereas those receiving an LVAD as destination therapy face a nearly 50% mortality rate [19,20]. Post-implantation complications are myriad and include bleeding, stroke, infection, pump thrombosis, and right ventricular failure [21].

Clinical risk factors for post-implantation complications and all-cause mortality remain under investigation. However, recent evidence indicates that elevated blood urea nitrogen (BUN) and a history of coronary artery bypass grafting or valve procedure reliably predict all-cause mortality [22]. In addition, psychosocial risk factors—including psychiatric illness and limited social support—portend an increased risk of post-implantation complications [23]. Predictors of all-cause mortality in the post-implantation setting include a reduced estimated glomerular filtration rate (eGFR) at hospital discharge, the development of VAs, and hemocompatibility-related adverse events during hospitalization [22].

Arrhythmias commonly occur in the post-implantation setting. VAs occur in approximately 20 to 50% of LVAD recipients; the arrhythmia burden tends to decline over the first several weeks and months following implantation [24,25]. Ventricular tachycardia (VT) and ventricular fibrillation (VF) frequently develop de novo in LVAD recipients with no prior history of arrhythmia [26]. New-onset VAs have been shown to significantly increase mortality in LVAD recipients [27,28,29]. In addition, up to 42% of patients with implantable cardioverter-defibrillators (ICDs) will experience shocks following LVAD placement, a significant proportion of which are delivered inappropriately. Increased ICD shocks have been associated with increased mortality, and thus the increased risk of ICD shocks following LVAD implantation has important clinical implications [30]. Risk factors for post-implantation VA include a preoperative history of VA, a preoperative history of atrial fibrillation, a history of nonischemic cardiomyopathy, or a history of end-stage renal disease (ESRD) [31,32,33].

Our findings are consistent with prior data and suggest that both VT and VF are associated with increased mortality in the post-implantation setting. In our cohort, the odds of mortality were higher among patients with VF relative to those with VT (aOR: 1.62 (CI: 1.38–1.91) and aOR: 1.30 (CI: 1.14–1.48), respectively). The mechanisms underlying post-implantation VA remain to be definitively established. A mechanical etiology mediated by excessive ventricular unloading has been proposed as a possible mechanism of pathogenesis for LVAD-associated VAs. In a 2007 study by Vollkron et al., the authors found that “suction events”—characterized by reduced pump filling with resultant negative left ventricular pressure—reliably induced VAs, including monomorphic VT, polymorphic VT, and VF [34]. It has also been proposed that mechanical changes in cardiac function directly related to LVAD placement may cause transient dysfunction in cardiomyocyte repolarization, as evidenced by a prolonged QTc interval in the immediate post-implantation period [35,36]. Non-mechanical mechanisms of VA include scar-related re-entry [37] and electrolyte shifts in the early post-implantation period [38]. Notably, both VAs and electrolyte disturbances—including hypo- and hyperkalemia—were associated with increased mortality in our cohort. It is therefore conceivable that the increased mortality observed among patients with electrolyte disturbances can be attributed to the resultant arrhythmia burden.

Management of VAs in LVAD recipients is controversial [24,39]. Ablation may be performed before, during, or after LVAD implantation; one small case series showed that concomitant LVAD implantation and cryoablation reduced the post-implantation arrhythmia burden [40]. However, peri-implantation ablation has also been shown to increase the risk of device thrombosis [41]. Post-implantation ablation may be technically challenging but can be effective in reducing or eliminating VAs in LVAD recipients [42].

The relationship between LVAD implantation and the risk of AAs remains incompletely understood. The data are inconsistent; several studies have shown that an existing diagnosis of atrial fibrillation is not associated with an increased risk of mortality [43,44,45], but at least two recent trials demonstrated that patients with atrial fibrillation faced a significantly increased risk of early right heart failure and death following LVAD implantation [46,47]. Our analysis revealed no significant association between AAs and mortality. The relationship between preimplantation AAs and mortality thus remains controversial. Atrial fibrillation may also develop de novo in the post-implantation setting and can occur in over 25% of LVAD recipients [48]. The development of atrial fibrillation in LVAD recipients has been associated with an increased risk of bleeding, prolonged ventilation, and worsened survival [47,48]. The mechanism by which LVAD implantation increases the risk of AAs is like that of VAs and may be related to left atrial suction events, left atrial remodeling, or elevated atrial pressures [49]. Management of post-implantation AAs may involve rate or rhythm control and anticoagulation. Although clinical practice guidelines remain to be established, a combination of a beta-blocker and digoxin has been shown to reduce mortality in HF patients with atrial fibrillation and may be indicated for LVAD recipients with AAs [50]. Systemic anticoagulation is nearly always administered to LVAD recipients to reduce the risk of pump thrombosis, and thus additional anticoagulation is not required for most patients with AAs. Catheter ablation may be appropriate for select patients with persistent atrial flutter; in one study, ablation resulted in immediate improvement in right heart failure among a small cohort of LVAD recipients [51]. In another case report, an LVAD recipient with poorly tolerated recurrent AAs was successfully treated with radiofrequency ablation [52]. However, although both pharmacologic therapy and ablation may be effective for some LVAD recipients, further investigation is required to establish optimal management strategies and develop validated clinical practice guidelines.

Metabolic derangements and electrolyte abnormalities can contribute to poor outcomes in LVAD recipients. In our cohort, acidosis and hyperkalemia were both associated with a significantly increased risk of mortality (aOR: 2.44 (CI: 2.14–2.77) and aOR: 1.54 (CI: 1.28–1.85), respectively). Acidosis is known to induce pulmonary vasoconstriction and increase peripheral vascular resistance, which decreases left ventricular filling and results in diminished LVAD flow [53]. Acidosis also represents a marker of end-organ ischemia or hypoperfusion, and thus increased mortality among patients with acidosis is not unexpected. Electrolyte abnormalities may contribute to increased mortality among LVAD recipients through two discrete mechanisms. First, hyperkalemia, hypokalemia, hypomagnesemia, hyponatremia, and hypocalcemia have all been independently associated with an increased risk of AAs and VAs [54,55,56]. Second, electrolyte imbalances may contribute to increased oxidative stress via dysregulation of various neurohormonal processes, which can have negative inotropic effects and cause cardiac remodeling [57]. Implantation of an LVAD frequently results in rapid electrolyte shifts due to sudden reperfusion of chronically hypoperfused tissues, altered renal excretion, and disruption of adaptive homeostasis [38,58]. Close monitoring and management of serum electrolytes is therefore essential to reduce the risk of post-implantation arrhythmias and improve patient outcomes. Interestingly, hypokalemia and hypomagnesemia represented protective factors in our analysis; although this appears paradoxical, clinical experience suggests that patients who are found to have electrolyte deficiencies on hospital admission receive prompt replacement therapy and are closely monitored on telemetry, allowing for early recognition of clinical deterioration and rapid intervention to prevent decompensation.

Females faced significantly higher odds of inpatient mortality as compared to males in our cohort. Sex differences in LVAD recipient mortality have previously been described in the medical literature. In a randomized–controlled trial by Hsich et al., the investigators found that female LVAD recipients faced an increased risk of neurologic events relative to their male counterparts [59]. A subsequent retrospective cohort study by Joshi et al. showed that the female sex was independently associated with increased post-implantation inpatient mortality during the era of pulsatile-flow devices but also reported that sex disparities in outcomes dissipated following the introduction of next-generation continuous-flow devices [60]. The Joshi group utilized data obtained from the National Inpatient Sample between 2004 and 2016, like our study. However, our analysis includes more recent data obtained between 2019 and 2020, and thus it is likely that most patients in our sample received continuous-flow LVADs. Nevertheless, in contrast to the results reported by Joshi et al., the odds of death among females in our cohort were 1.4 times higher than that of males (95% CI: 1.25–1.59). Other more recent studies have reported similar results; in a 2023 retrospective cohort study that included nearly 20,000 LVAD recipients, Shetty et al. found that females faced a significantly higher rate of adverse events and a higher risk of all-cause mortality relative to males across all sociodemographic subgroups [61]. The authors hypothesized that the outcome disparities could largely be attributed to social determinants of health, noting that females classically assume the role of caregiver, and thus females with HF are less likely to receive adequate family or spousal support and timely medical management [62]. The authors also observed that females had a significantly increased risk of bleeding and stroke relative to males and postulated that sex differences in coagulofibrinolytic responses could contribute to disparate outcomes [63].

A few additional factors were associated with an increased mortality risk in our cohort. Increasing age was independently associated with an increased risk of death: for every unit increase in age, the odds of inpatient mortality were 1.02 times higher. The medical literature has well-established the effect of increasing age on inpatient mortality in LVAD recipients [64,65]. Complete heart block was also associated with significantly higher odds of mortality, which may be related to decreased LVAD flow and right ventricular failure [66,67]. Lastly, invasive mechanical ventilation was associated with significantly increased mortality among LVAD recipients, which likely reflects the baseline disease severity of patients requiring mechanical ventilation rather than a distinct pathophysiologic process unique to mechanical ventilation in the setting of LVAD implantation [13].

There are a few limitations to our study inherent to its design. First, the study was retrospective, and thus causality cannot be assessed. Second, the NIS database does not include specific data about the make, model, or generation of LVAD used in each encounter; it is uncertain how many patients received pulsatile- versus continuous-flow devices. Third, the NIS database does not distinguish between patients undergoing LVAD implantation as a bridge versus destination therapy, limiting our ability to perform a subgroup analysis. Fourth, the NIS database does not include data on the etiologies of diseases, the chronicity of diseases (except when captured in the specific ICD-10 nomenclature), medications used by the subjects, or laboratory values. We were therefore unable to include these data or control for them in our analyses. Nevertheless, despite these limitations, our study provides important insight into pre- and post-implantation LVAD risk factors and should prompt further investigation into optimal management strategies.

## 5. Conclusions

Our analysis of nearly 10,000 hospitalizations with LVAD recipients establishes that acid–base imbalance, electrolyte abnormalities, and VAs increase the risk of inpatient mortality. The results of our analysis suggest that close monitoring with aggressive electrolyte and acid–base management can help improve patient outcomes. In addition, pharmacological treatment or ablation of VAs may reduce the risk of death in the appropriate patient population. Our analysis also revealed persistent gender disparities in LVAD recipient outcomes, with females facing a higher burden of post-implantation complications and death relative to males. Addressing social determinants of health and identifying underlying physiologic differences may help improve outcomes among female LVAD recipients in both the pre- and post-implantation settings. Future research is warranted to further understand the effect of AAs on LVAD recipient outcomes and develop strategies to reduce mortality among at-risk patients.

## Figures and Tables

**Table 1:** Comparison of subject demographics and chronic comorbid conditions of LVAD patients who survived versus those died during the hospital stay. Key: ** = *p*-value < 0.001; * = *p* < 0.01.

	LVAD (9418)		
	Survived (6879 (73%))	Died (2539 (27%))	*p*-Value
**Age Group (years)**	n (%)	n (%)	**
18–50	904 (13.1)	227 (8.9)	
51–60	1386 (20.2)	409 (16.1)	
61–70	1991 (28.9)	787 (31.0)	
71–80	1683 (24.5)	784 (30.9)	
81–90	915 (13.3)	332 (13.1)	
**Sex**			**
Female	1800 (26.2)	834 (32.8)	
Male	5079 (73.8)	1705 (67.2)	
**Race**			*
White	4818 (70.0)	1817 (71.6)	
Black	935 (13.6)	282 (11.1)	
Hispanic	593 (8.6)	217 (8.5)	
Asian or Pacific Islander	210 (3.1)	93 (3.7)	
Native American	56 (0.8)	24 (0.9)	
Other	267 (3.9)	106 (4.2)	
**Chronic Comorbid Conditions**			
Alcohol abuse	263 (3.8)	70 (2.8)	0.67
Chronic pulmonary disease	1383 (20.1)	449 (17.7)	0.26
Diabetes	3126 (45.4)	1078 (42.5)	0.09
Drug abuse	175 (2.5)	48 (1.9)	0.79
HIV/AIDS	29 (0.4)	13 (0.5)	0.97
Hypertension	4520 (65.7)	1615 (63.6)	0.13
Liver Disease	197 (2.9)	52 (2.0)	0.74
Leukemia	36 (0.5)	18 (0.7)	0.92
Lymphoma	48 (0.7)	25 (0.9)	0.92
Metastatic Cancer	44 (0.6)	23 (0.9)	0.9
Obesity	1480 (21.5)	480 (18.9)	0.22
Peripheral vascular disease	1140 (16.6)	419 (16.5)	0.97
Chronic kidney disease	2542 (36.9)	858 (33.8)	0.09
Rheumatoid diseases	137 (12.0)	55 (2.2)	0.93
Smoking, current and history of	2360 (34.3)	690 (27.2)	**
**Charlson Comorbidity Index**			0.07
0	121 (1.8)	28 (1.1)	
1	788 (11.4)	303 (11.9)	
≥2	5970 (86.8)	2208 (87.0)	

## Data Availability

Publicly available datasets were analyzed in this study. This data can be found at the distributor site here: https://cdors.ahrq.gov/, accessed on 4 March 2024.
